# Negative regulation of cationic nanoparticle-induced inflammatory toxicity through the increased production of prostaglandin E2 via mitochondrial DNA-activated Ly6C^+^ monocytes: Erratum

**DOI:** 10.7150/thno.37524

**Published:** 2019-07-17

**Authors:** Li Liu, Yantong Liu, Bocheng Xu, Chuyu Liu, Yanpeng Jia, Ting Liu, Chunju Fang, Wei Wang, Jun Ren, Zhiyao He, Ke Men, Xiao Liang, Min Luo, Bin Shao, Ye Mao, Henyi Xiao, Zhiyong Qian, Jia Geng, Birong Dong, Peng Mi, Yu Jiang, Yuquan Wei, Xiawei Wei

**Affiliations:** Lab of Aging Research and Nanotoxicology, State Key Laboratory of Biotherapy, West China Hospital, Sichuan University and Collaborative Innovation Center, No. 17, Block 3, Southern Renmin Road, Chengdu, Sichuan 610041, PR China

In our paper 1, the Supplementary Figure 10 and Supplementary Figure 16C contain errors. In Supplementary Figure 10 and 16C the “NS” column was inadvertently duplicated for the “NS” images in Figure 1B in the main text. The correct Supplementary Figure 10 and Supplementary Figure 16 appear below in the supplementary material. We would like to apologize for this inconvenience.

## Supplementary Material

Supplementary figures.
