# Supplementary material for: Negative regulation of cationic nanoparticle-induced inflammatory toxicity through the increased production of prostaglandin E2 via mitochondrial DNA-activated Ly6C+ monocytes: Erratum
Source: Theranostics. 2019 Jul 17;9(19):5443. doi: 10.7150/thno.37524 (PMC6735400; doi:10.7150/thno.37524)
Supplement: Supplementary file 1 — Supplementary figures. [file thnov09p5443s1.pdf]

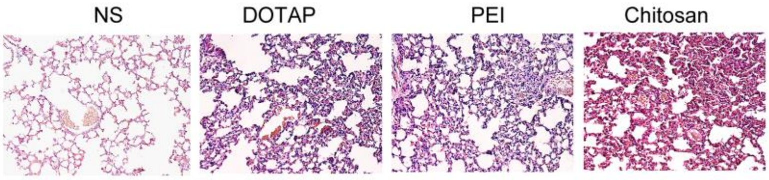

Supplementary Figure 10. Representative H&E stained section of lung from the mice treated with DOTAP liposomes (25 mg/kg), PEI (5 mg/kg) and chitosan (25 mg/kg) after 24 h (n=5), scale bars=50μm. Data are representative of three independent experiments.

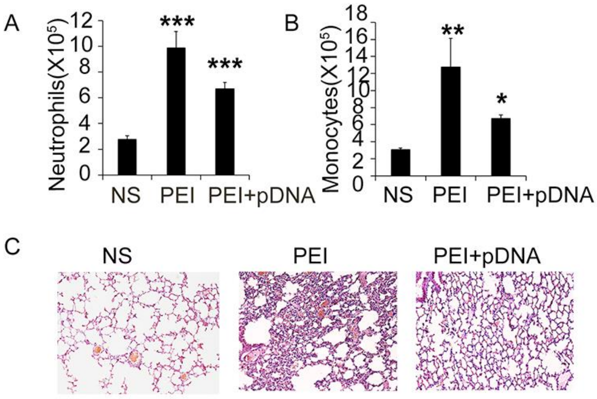

Supplementary Figure 16. C57BL/6 mice were treated with intravenous injection of PEI nanoparticles (5 mg/kg), PEI/pVAX plasmid lipoplexes (w/w 5/5) or normal saline as control. Flow cytometry analysis of recruited cells in lung after administration for 24 h. Numbers indicate either $CD45^+CD11b^+Ly6C^+$ monocytes or $CD45^+CD11b^+Ly6G^+$ neutrophils in the quadrant expressed as number of the cells in the lung after perfusion. (n=5). And Representative H&E stained section of lung from the mice treated with PEI nanoparticles (5 mg/kg), PEI/pVAX plasmid lipoplexes (w/w 5/5) or normal saline as control. Data are representative of three independent experiments, and the results are expressed as the means ± S.E.M. Statistical comparisons were performed using Student's t-test or Dunnet's t-test (**P*<0.05; ***P*<0.01; ****P*<0.005).
